# Cost-effectiveness analysis of isavuconazole versus voriconazole for the treatment of patients with possible invasive aspergillosis in Sweden

**DOI:** 10.1186/s12879-019-3683-2

**Published:** 2019-02-11

**Authors:** Lefteris Floros, Daniel Kuessner, Jan Posthumus, Emma Bagshaw, Jan Sjölin

**Affiliations:** 1 Covance Market Access, London, UK; 2 Basilea Pharmaceutica International Ltd, Basel, Switzerland; 3 Department of Medical Sciences, Infectious Diseases, Uppsala University, Uppsala, Sweden; 4 Covance Market Access, London, UK

**Keywords:** Isavuconazole, Voriconazole, Cost-effectiveness, Invasive aspergillosis, Mucormycosis, Sweden, Antifungal

## Abstract

**Background:**

Voriconazole is well established as standard treatment for invasive aspergillosis (IA). In 2017, isavuconazole, a new antifungal from the azole class, with a broader pathogen spectrum, was introduced in Sweden. A model has therefore been developed to compare the cost-effectiveness of isavuconazole and voriconazole in the treatment of possible IA in adults in Sweden.

**Methods:**

The cost-effectiveness of isavuconazole versus voriconazole was evaluated using a decision-tree model. Patients with possible IA entered the model, with 6% assumed to actually have mucormycosis. It was also assumed that pathogen information would become available during the course of treatment for only 50% of patients, with differential diagnosis unavailable for the remainder. Patients who were considered unresponsive to first-line treatment were switched to second-line treatment with liposomal amphotericin-B. Data and clinical definitions included in the model were taken from the published randomised clinical trial comparing isavuconazole with voriconazole for the treatment of IA and other filamentous fungi (SECURE) and the single-arm, open-label trial and case-control analysis of isavuconazole for the treatment of mucormycosis (VITAL). A probabilistic sensitivity analysis was used to estimate the combined parameter uncertainty, and a deterministic sensitivity analysis and a scenario analysis were performed to test the robustness of the model assumptions. The model followed a Swedish healthcare payer perspective, therefore only considering direct medical costs.

**Results:**

The base case analysis showed that isavuconazole resulted in an incremental cost-effectiveness ratio (ICER) of 174,890 Swedish krona (SEK) per additional quality adjusted life-year (QALY) gained. This was mainly due to the efficacy of isavuconazole against IA and mucormycosis, as opposed to voriconazole, which is only effective against IA. Sensitivity and scenario analyses of the data showed that the average ICER consistently fell below the willingness to pay (WTP) threshold of 1,000,000 SEK. The probability of isavuconazole being cost-effective at a WTP of 170,000 SEK per QALY gained was 50% and at a WTP of 500,000 SEK per QALY gained was 100%.

**Conclusions:**

This model suggests that the treatment of possible IA with isavuconazole is cost-effective compared with treatment with voriconazole from a Swedish healthcare payer perspective.

**Electronic supplementary material:**

The online version of this article (10.1186/s12879-019-3683-2) contains supplementary material, which is available to authorized users.

## Background

Invasive fungal diseases (IFDs) are a common complication among immunocompromised patients, such as patients with haematological malignancies, haematopoietic stem cell transplant (HSCT) recipients, solid organ transplant recipients and patients in intensive care units [1]. IFDs such as invasive aspergillosis (IA) or mucormycosis are also associated with substantial morbidity and high mortality, especially in high-risk groups [2]. Both IFDs are relatively rare; for example, in an epidemiology study in France, 35,876 patients with IFDs were identified between 2001 and 2010, with an incidence of 1.4 cases/100,000 general population/year for IA and 0.09 cases/100,000 general population/year for mucormycosis [3].

Treatment of IFDs is often initiated before confirmation of the causative pathogen, based on patient risk factors and clinical and radiological signs. In the case of IA, disease is classified as proven, probable or possible IA based on criteria from the European Organisation for Research and Treatment of Cancer/Invasive Fungal Infections Cooperative Group and the National Institute of Allergy and Infectious Diseases Study Group [4]. In the clinical setting, diagnosis of IA is difficult, as its symptoms are not specific, and the fungus can be found in the airways of healthy individuals [5, 6]. Diagnosis of mucormycosis is even more challenging due to its rarity and the lack of a rapid diagnostic test, and because clinical and radiological presentations resemble those of IA [7]. Additionally, mucormycosis can occur as a co-infection with IA, complicating the chance of differential diagnosis between the two diseases [8]. Therefore, most cases of mucormycosis will initially be presumed to be IA until a definite diagnosis is confirmed. However, based on the 10-year trend of IFDs in France [3], it can be assumed that approximately 6% of patients diagnosed with possible IA would actually have mucormycosis. Although epidemiological data for Sweden are lacking, a single-centre, retrospective observational study of 100 patients with proven or probable invasive mould disease in Sweden reported that 14% of isolates were Mucorales spp. [9]. Making a differential diagnosis between the two diseases is important, as delaying treatment of mucormycosis by ≥6 days has been reported to substantially increase mortality [10].

Isavuconazonium sulfate, the prodrug of isavuconazole, is the most recently available antifungal triazole drug approved by the US Food and Drug Administration (FDA) for the treatment of adults with IA or invasive mucormycosis, and by the European Medicines Agency (EMA) including Sweden for the treatment of adults with IA or the treatment of mucormycosis when amphotericin B (AmB) is not appropriate. Approvals were based on the results of the SECURE trial, which included a comparator group (voriconazole) [11], and a case-control analysis between the single-arm VITAL study and the Fungiscope™ registry (comparison between isavuconazole and AmB) [12], as well as comparisons to historical data from patients with untreated mucormycosis or delayed treatment [10, 13].

Voriconazole has long been the standard of care in the treatment of IA, but treatment guidelines also now include recommendations for isavuconazole for the primary treatment of IA [14, 15]. Since isavuconazole and voriconazole each have different spectra of antifungal activity and safety profiles, it is important to determine their relative cost effectiveness. From a US hospital perspective, isavuconazole has been demonstrated to be a cost-effective treatment for IA in hospitalised patients compared with voriconazole [16]. However, the cost-effectiveness of isavuconazole compared with voriconazole in European countries has not been reported. Therefore, a cost-effectiveness model was designed to investigate the economic value to the Swedish healthcare system of introducing isavuconazole for the treatment of possible IA in adults when, at the point of treatment initiation, a differential diagnosis between IA and mucormycosis had not been achieved.

## Methods

### Clinical context and key assumptions

The model was developed to present a cost-utility analysis of isavuconazole versus voriconazole in the treatment of patients with possible IA. It was assumed that antifungal treatment was initiated before pathogen information was available to clinicians, and that this information would become available during the course of treatment for only 50% of patients (base case), while differential diagnosis would not be achieved for the remainder. The causative pathogen was, in most cases, an *Aspergillus* species (94% of patients in the base case) and a small fraction, Mucorales (6%). The clinical outcomes were assigned based on the clinical data related to the true underlying pathogens and treatment group. The source of the clinical data was the SECURE trial (comparison between isavuconazole and voriconazole [11], and a case-control analysis between the single-arm VITAL study and the Fungiscope™ registry (comparison between isavuconazole and AmB) [12], as well as comparisons to historical data from patients with untreated mucormycosis or delayed treatment [10, 13]. These sources were part of the clinical review of isavuconazole by the FDA and EMA (Table 1). For second-line treatment we assumed that the resource use (e.g., treatment duration) was as per first-line therapy. The model followed a Swedish healthcare payer perspective, and therefore only considered direct medical costs.

**Table 1:** Data sources for all-cause mortality at day 84

Treatment group	Invasive aspergillosis	Mucormycosis
Isavuconazole	29% (SECURE trial: Randomized controlled trial [11]^a^)	43% (VITAL trial: Open-label study [12])
Voriconazole	29% (SECURE trial: Randomized controlled trial	Patients with delayed treatment^b^: 83% (retrospective observational study [10]^c^)
		No (effective) treatment^d^: 96% (meta-analysis of untreated patients [27]^e^)

*L-AmB* liposomal amphotericin B

^a^Assumed to be the same for isavuconazole and voriconazole based on lack of significant difference in the SECURE trial

^b^In model: patients treated with voriconazole followed by L-AmB due to mucormycosis identification

^c^All-cause mortality at day 84 of patients with treatment delayed by more than 6 days

^d^In model: patients receiving voriconazole who remained undiagnosed

^e^Meta-analysis based on retrospective reviews [30, 43] and Fungiscope™ database

### Model development and structure

**Fig. 1:**
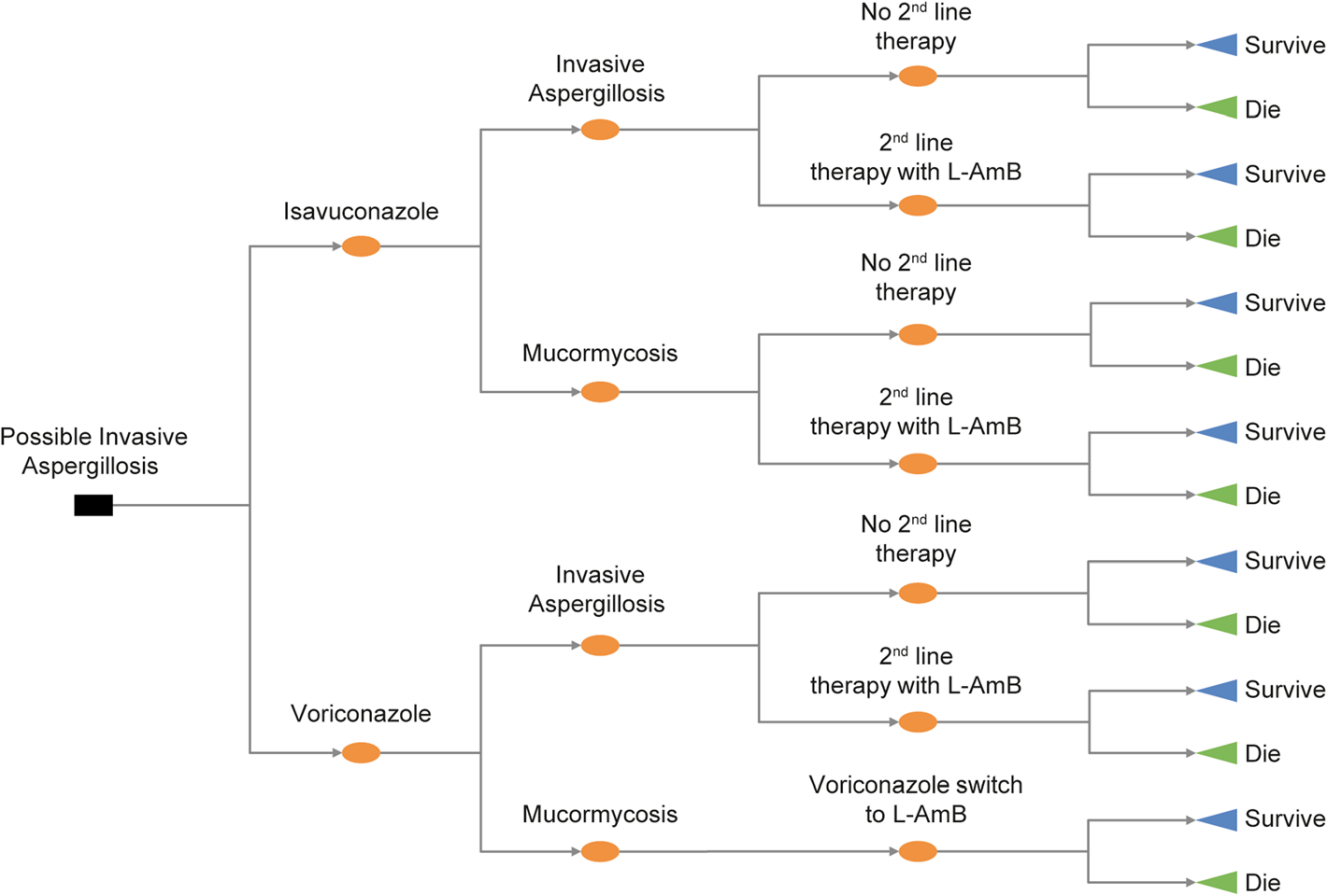
Decision-tree model structure. First-level decision nodes represent the treatment comparison. Second-level decision nodes represent the IA/mucormycosis pathogen split. Third-level decision nodes are associated with second-line treatment options. IA, invasive aspergillosis; L-AmB, liposomal amphotericin-B

A decision-tree approach was used to represent the short-term patient pathway, with branches representing either the presence of IA or mucormycosis from initial symptoms to outcome after antifungal treatment (completion of antifungal treatment on resolution of infection or death; Fig. 1). Patients entered the model with possible IA and treatment was initiated with either isavuconazole or voriconazole. Patients in both treatment groups with IA (irrespective of whether it was confirmed) could either remain on first-line treatment, or if not responding to initial treatment, could switch to second-line treatment with liposomal amphotericin B (L-AmB). For isavuconazole-treated patients with mucormycosis, it was assumed that, irrespective of whether the pathogen was identified during the treatment course, treatment would continue for a duration appropriate for mucormycosis. Similarly as for IA, patients with mucormycosis could switch to second-line treatment with L-AmB. Voriconazole-treated patients for whom mucormycosis was subsequently confirmed would be switched to treatment with L-AmB. Voriconazole-treated patients with mucormycosis that remained unidentified would continue receiving treatment as appropriate for IA with the consequence of not being correctly treated.

Results were extrapolated to a lifetime horizon using the average life expectancy and quality of life relevant to the underlying health conditions of patients treated for IFDs. This approach follows another cost-effectiveness model study [17].

### Model inputs

#### Treatment regimens and dosing

##### First line treatment

Dosing schedules for isavuconazole and voriconazole were according to the SECURE study and their respective summary of product characteristics (SmPC) (Table 2) [11, 18–20]. Weight-based dosing (e.g. voriconazole IV) was calculated using the mean weight of 71.4 kg (standard deviation [SD]: 16.4 kg) observed for European patients in the SECURE study. In both treatment groups, it was assumed that 75% of patients started on intravenous (IV) treatment and 25% of patients started on oral treatment, based on data from the IA subgroup from VITAL [12, 21]. In a scenario analysis, the effects of assuming that 100% of patients started on IV treatment were also tested.

**Table 2:** Resource use and acquisition costs

Resource use item	Costs (SEK)
Drug costs^a^	
-Isavuconazole^b^ (200 mg vial), 200 mg/day (maintenance dose)	5311
-Isavuconazole^b^ (14-pack of 100 mg capsules), 200 mg/day (maintenance dose)	7067
-Voriconazole^c^ (200 mg vial), 2 × 4 mg/kg/day (maintenance dose)	1167
-Voriconazole^d^ (56-pack of 200 mg capsules), 400 mg/day (maintenance dose)	8008
-L-AmB (50 mg vial, 10 vials), 5 mg/kg/day	15,869
-Posaconazole^e^ (96-pack of 100 mg tablets), 300 mg/day	27,938
Hospitalisation costs/day^f^	7006
Monitoring cost/unit^g^	611
Adverse event costs by system order class/event	
-Cardiac (i.e., cardiac arrest, tachycardia)^h^	18,966
-Hepatobiliary (i.e., hyperbilirubinemia, abnormal hepatic function, jaundice, cholestasis)^i^	3863
-Nephrotoxicity^j^	89,316

*L-AmB* liposomal amphotericin B, *SEK* Swedish krona

^a^Drug retail prices were taken from the TLV price list for oral isavuconazole, oral voriconazole (generic), liposomal amphotericin-B and posaconazole [27]; the retail prices of IV isavuconazole and voriconazole (generic) were taken from Apoteket.se [28] (March 2018)

^b^IV and oral isavuconazole loading dose was 200 mg every 8 h on days 1 and 2; maintenance dose was initiated from day 3 onwards

^c^IV voriconazole loading dose was 6 mg/kg every 12 h on day 1, maintenance dose was initiated from day 2 onwards

^d^Oral voriconazole loading dose was 400 mg/kg on day 1, maintenance dose was initiated from day 2 onwards

^e^Posaconazole loading dose was 300 mg twice daily on day 1, maintenance dose was initiated from day 2 onwards

^f^Costs taken from South Sweden Price list [29]; Inpatient daily cost at haematology clinic. Code: VD010 (p. 50)

^g^Cost of liver function test taken from South Sweden Price list [29]

^h^Costs taken from South Sweden Price list [29]: Cardiac arrest uncomplicated. Code: E48E: 43,228 SEK (29%) and tachycardia: Arrhythmia uncomplicated inpatient [Code: E65E) 8976 SEK (71%)

^i^Costs taken South Sweden Price list [29]: Physician visit liver/gallbladder. Code: G99O 3863 SEK

^j^Costs taken from South Sweden Price list [29]: Inpatient day at nephrology dept. Code: VD010 9924 SEK. Based on Bruynesteyn et al., 2007, 9 incremental days due to nephrotoxity were applied

For the first-line treatment of IA, the treatment durations were according to the SECURE study (47.1 days) [11, 22]. For treatment of mucormycosis, the treatment duration of isavuconazole was set to the treatment duration observed in VITAL (149.0 days) [12]. Patients with mucormycosis receiving voriconazole for whom pathogen information became available were switched to second-line treatment after day 11, based on the median time of 11 days (range 2–34 days) between clinical signs and diagnosis of mucormycosis in a French nationwide retrospective study evaluating the clinical characteristics and outcomes of mucormycosis in allogeneic HSCT patients [23]. For patients with mucormycosis treated with voriconazole who did not receive a diagnosis, treatment was assumed to proceed as per that for IA (Table 3).

**Table 3:** Treatment duration, and length of hospital stay

	Treatment	Resource use (days)	Source
*Treatment duration*			
First-line treatment for IA	Isavuconazole	47.1 (IV: 8.1)	Mean treatment duration from SECURE [11, 39]^a^
	Voriconazole	47.1 (IV: 8.1)	
First-line treatment for mucormycosis	Isavuconazole Voriconazole no pathogen information available Voriconazole non-responders prior to switching	149.0 (IV: 15.5) 47.1 (IV: 8.1) 11.0 (IV: 8.1)	Mean treatment duration from VITAL [12, 36] Patients treated as IA patients, assumption follows SECURE [11, 39] Median time between first clinical signs and mucormycosis diagnosis, Xhaard et al. [17]
Second-line treatment for IA	L-AmB	14.5 (IV: 14.5)	Median treatment duration from Leenders et al. [19]
	Oral step-down with voriconazole or posaconazole	32.6 (IV: 0)	Total duration of second-line treatment ^b^ minus duration of treatment with liposomal amphotericin-B
Second-line treatment for mucormycosis	L-AmB	27.2 (IV: 27.2)	Median treatment duration from VITAL Fungiscope case-control [12, 36]
	Posaconazole	121.8 (IV: 0)	Total duration of second-line treatment^c^ minus duration of treatment with liposomal amphotericin-B
*Length of hospital stay*			
IA	Isavuconazole Voriconazole L-AmB	19.7 19.7 19.7	Mean duration from SECURE [11, 39]^a^
Mucormycosis	Isavuconazole	19.3	Mean duration from VITAL [12, 36]
	Voriconazole no pathogen information available	19.3	Assumption same as for isavuconazole
	Voriconazole non-responders prior to switching	11.0	Duration of treatment before mucormycosis diagnosis from Xhaard et al. [17]
	L-AmB + posaconazole	27.2	Assumed: Duration the same as the mean days of IV treatment in Fungiscope case-control (VITAL [12, 36])

^a^As there was no statistical difference between treatment groups in SECURE study, the same value was taken for isavuconazole, voriconazole and L-AmB

^b^The total duration of second-line treatment for IA was assumed to be the equivalent to that for isavuconazole that for first-line treatment for IA (47.1 days)

^c^The total duration of second-line treatment for mucormycosis was assumed to be the equivalent to that for first-line treatment for mucormycosis with isavuconazole (149.0 days). IA, invasive aspergillosis; IV, intravenous; L-AmB, liposomal amphotericin B

##### Second line treatment

Based on data from the SECURE and VITAL trials, it was calculated that approximately 48% of patients with IA and 33% of patients with mucormycosis would require second-line treatment with L-AmB due to insufficient response to first-line treatment, based on the numbers who discontinued first-line treatment minus those who died during treatment. For patients treated initially with either isavuconazole or voriconazole (only IA), it was assumed that non-responders would switch to second-line treatment between 7 and 14 days (10.5 days). For patients treated with voriconazole and after achieving diagnosis of mucormycosis, it was assumed that the switching occurred after 11 days.

L-AmB was used in the model as a second-line treatment for both IA and mucormycosis after the failure of both first-line treatments (isavuconazole and voriconazole). L-AmB was dosed at 5 mg/kg for both IA and mucormycosis (based on current clinical practice in Sweden for IA, and on the FungiScope™ case-control study for mucormycosis [12, 24]). For patients with IA, it was assumed L-AmB would be followed by oral step-down treatment with either voriconazole (dosed as per SmPC [18]) for patients with IA if the fungal burden was reduced, e.g., due to the L-AmB treatment, or posaconazole tablets (dosed as per SmPC [25]) in a 1:1 ratio and the total duration (L-AmB and oral step-down treatment) was assumed to be equal to the first-line treatment duration (47.1 days), following an approach applied in a previous economic model [17]. The duration of L-AmB for patients with IA was based on a clinical study by Leenders et al. in which the median duration of treatment was 14.5 days [26]. The duration of second-line step-down treatment for IA patients with posaconazole or voriconazole was calculated as the difference between 14.5 days and the total duration of second-line treatment (47.1 days).

For patients with mucormycosis, treatment with L-AmB was based on the matched patients from FungiScope™ case-control study (27.2 days) [12] and the second-line step-down treatment was posaconazole (121.8 days, following the same estimation approach as in IA) (Table 3).

### Costs of drug acquisition, hospitalisation, adverse events, and outpatient monitoring

Drug retail prices were sourced from the Tandvards och Lakemedelsformansverket Verket (TLV) price list [27] (reimbursed formulations) and Apoteket.se [28] (non-reimbursed formulations). The cost/day of hospitalisation was calculated as 7006 Swedish krona (SEK), based on the daily rate reported in the South Sweden price list for inpatient stay at a haematology clinic [29], and was assumed to include costs associated with IV drug preparation and administration as well as monitoring during the hospital stay (Table 2).

For patients with IA, the mean duration of the initial hospital stay was based on the duration in the SECURE trial, which was not significantly different between treatment groups (Table 3) [11, 22]. Patients on second-line treatment with L-AmB followed by voriconazole or posaconazole were assumed to have the same length of hospital stay as those undergoing first-line treatment. Patients receiving second-line treatment would incur a proportion of first-line treatment costs plus the full costs of second-line treatment. For isavuconazole-treated patients with mucormycosis, a mean duration of the initial hospital stay was based on data from the VITAL trial (Table 3) [12]. Patients treated with voriconazole for whom pathogen information became available, were assumed to be hospitalised for the 11 days prior to switching to L-AmB followed by posaconazole treatment. For patients receiving second-line treatment with L-AmB followed by posaconazole, the mean duration of hospital stay was as per the duration of IV treatment observed in the FungiScope™ case-control study [12]. Patients treated with voriconazole for whom no pathogen information became available were assumed to be hospitalised as per the patients with IA (Table 3). Costs for surgical debridement or other measures in patients with mucormycosis were not included because data are lacking and would not have resulted in between-group differences.

The model estimated the cost of moderate/severe adverse events (AEs) of the cardiac and hepatobiliary system organ classes, since there was a significant difference between isavuconazole and voriconazole in either the proportion of patients affected or the overall number of AEs experienced in these classes in the SECURE trial [11]. The total number of moderate/severe AEs classed under cardiac disorders in the isavuconazole versus voriconazole groups was 28 of 257 patients and 47 of 259, respectively, and the number of moderate/severe AEs classed as hepatobiliary disorders was 17 of 257 and 37 of 259, respectively. These AEs would be expected to have substantial economic consequences (Table 2). For L-AmB-treated patients, the cost of treating nephrotoxicity was included, based on its high incidence in clinical trials [11] and its inclusion in previously published cost-effectiveness models [17, 30, 31]. Incidence of nephrotoxicity in patients treated with L-AmB was 11.5% based on the clinical trial by Walsh et al. 2004 [32]. Costs associated with each AE were sourced from the South Sweden price list [29] (Table 2).

For all patients, the duration of outpatient monitoring was based on total treatment length minus the length of hospitalisation. Based on the products’ drug labelling and differences in hepatotoxicity, the costing for liver function tests per treatment course was calculated (Additional file 1: Table S1). Based on the South Sweden price list [29], the cost of a liver function test of 611 SEK was applied (Table 2).

### Time horizon, discount rate and cost-effectiveness threshold

To capture the long-term effects of isavuconazole and voriconazole within the model, a lifetime horizon was used. A discount rate of 3% was applied based on the requirements of the TLV. A 1000,000 SEK per additional quality-adjusted life year (QALY) willingness to pay (WTP) threshold was applied.

### Utility and life expectancy

It was assumed that patients surviving an IFD would experience the quality of life and life expectancy associated with their underlying condition. The most common underlying disease for patients in SECURE and VITAL was acute myeloid leukaemia (AML) therefore the base case analysis used a utility figure of 0.82, based on an analysis of AML survivors [33]. A life expectancy of 17 years was sourced from survival trends of AML patients in Sweden from 1973 to 2011 [34] and this was further discounted using a 3% rate and value factor sum method to 13.6 years. Both utility and life expectancy values were tested in the deterministic sensitivity analysis and probabilistic sensitivity analysis.

### All-cause mortality (ACM)

Table 1 lists the data sources (SECURE, VITAL and comparisons to historical data from patients) which were used to populate the model. An ACM through day 84 of 29% for patients with IA was used in this analysis, as per the isavuconazole arm of the SECURE trial (not significantly different between treatment groups). This percentage was also used for second-line treatment of patients with L-AmB without applying a mortality figure for first-line treatment (to avoid double counting). For patients treated with isavuconazole for mucormycosis, an ACM at day 84 of 43% was used, based on the results of the VITAL trial [12]. For patients treated with voriconazole with mucormycosis for whom pathogen information became available (i.e., switched to L-AmB at day 11 [23]), an ACM at day 84 of 83% was used, based on the mortality rate observed in a study of mucormycosis that included patients who received delayed effective treatment [10]. For patients with mucormycosis treated with voriconazole for whom no pathogen information became available, an ACM at day 84 of 96% was used, based on the mortality rate observed in a meta-analysis of untreated mucormycosis patients [13].

### Sensitivity/scenario analyses and estimation of cost-effectiveness

In probabilistic sensitivity analyses, a probability distribution for each input parameter (mucormycosis prevalence, pathogen identification percentage, second-line treatment drug costs, percentage receiving second-line treatment and percentage of patients requiring therapeutic drug monitoring) was defined to account for the uncertainty around the input point estimates. During every run, a value for each of the inputs was randomly selected simultaneously from its probability distribution and used to calculate mean costs and mean QALYs. The probabilistic variables and their distributional parameters are summarised in Additional file 2: Table S2. The model was run 1000 times and mean costs and QALYs were summarised. To test the robustness of model assumptions, a deterministic sensitivity analysis was also performed for a number of variables in the model (Additional file 3: Table S3), and a scenario analysis was performed to test how certain parameters affected the model. The incremental cost-effectiveness ratio (ICER) was calculated using the equation:

$$ ICER=\frac{Costs(B)- Costs(A)}{QALYs(B)- QALYs(A)} $$

Isavuconazole was considered cost-effective if the ICER was less than the previously defined WTP threshold.

## Results

### Base case analysis

Isavuconazole was both more costly and more effective than voriconazole (Table 4). Isavuconazole resulted in 0.3 more QALYs per patient than voriconazole at an incremental cost of 52,191 SEK, resulting in an ICER of 174,890 SEK per additional QALY gained. Drug acquisition costs were 51% higher for isavuconazole compared with voriconazole, but voriconazole had higher treatment-emergent adverse event (TEAE) costs (24%) and drug monitoring costs (34%). Overall hospitalisation costs were comparable between arms.

**Table 4:** Base case analysis results

	Costs (SEK)					Effects	
	Drug	Hospital	AEs	Monitoring	Total costs	LYs	QALYs
Isavuconazole							
IA^a^	143,966	192,098	6819	1690	344,573	9.07	7.43
Mucormycosis^a^	18,489	11,427	331	307	30,554	0.45	0.37
Combined	162,455	203,525	7150	1996	375,127	9.51	7.80
Voriconazole							
IA^a^	96,872	192,098	8395	2617	299,982	9.07	7.43
Mucormycosis^a^	10,877	11,582	438	58	22,954	0.08	0.07
Combined	107,749	203,680	8832	2675	322,936	9.15	7.50
Difference (isavuconazole−voriconazole)					52,191		0.3
						ICER (SEK/ QALY) 174,890	

*AE* adverse event, *IA* invasive aspergillosis, *ICER* incremental cost-effectiveness ratio, *LY* life year, *QALY* quality-adjusted life year, *SEK* Swedish krona

^a^The costs and effects for IA and mucormycosis are weighted by their relative proportions, e.g., 94% for IA and 6% for mucormycosis

### Sensitivity and scenario analyses

**Fig. 2:**
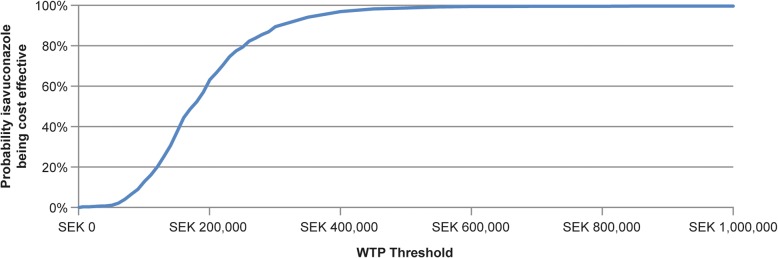
Isavuconazole – cost-effectiveness acceptability curve from the probabilistic sensitivity analysis. QALY, quality-adjusted life-year; WTP Threshold, threshold of willingness to pay cost per QALY

**Fig. 3:**
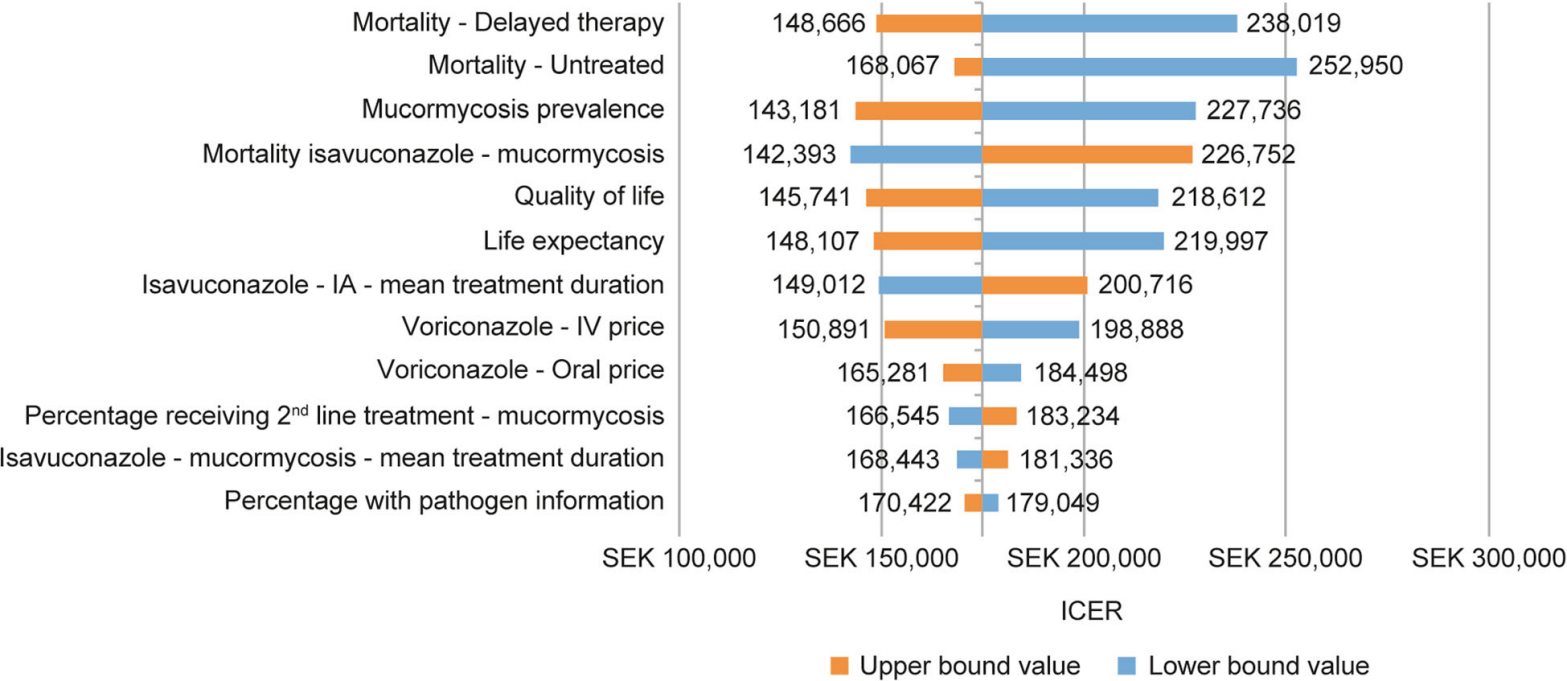
Deterministic sensitivity analysis of results^a^. ^a^All variables were tested with an upper and lower bound value of + 25 and − 25%, respectively, except for mortality – delayed therapy with + 20 and − 30%, mortality untreated with + 4 and − 30%, and quality of life with + 20 and − 20%, with upper and lower bound values, respectively. IA, invasive aspergillosis; ICER, incremental cost-effectiveness ratio; IV, intravenous; SEK, Swedish krona

In probabilistic sensitivity analyses, after 1000 runs in which estimates of variations in costs, percentages requiring second-line treatment, mortality, and quality of life were randomly chosen, the average ICER consistently fell below the WTP threshold of 1000,000 SEK. The probability of isavuconazole being cost-effective at a WTP threshold of 175,000 SEK per QALY gained was 50% and at a WTP threshold of 550,000 SEK per QALY gained was 100% (Fig. 2). Figure 3 shows 12 parameters that were shown to have most influence on the model results (of the 16 parameters tested). The results were most sensitive to changes in mortality figures. Reducing mortality by 30% in patients receiving delayed L-AmB treatment or no effective treatment resulted in an increase of the ICER to 238,019 SEK and 252,950 SEK, respectively. Varying mucormycosis prevalence or life expectancy by 25%, or varying quality of life by 20%, also had a measurable effect on the model results. Various parameters were also tested in scenario analyses to determine their impact on the results of the model (Table 5). Using a higher mucormycosis prevalence of 14% (obtained from a Swedish study [9]), the ICER was reduced by 53% to 81,464 SEK. When the proportion of patients for whom pathogen information became available was set at 0%, the ICER was increased by approximately 15,000 SEK, and when that proportion of patients was set at 100%, the ICER was decreased by approximately 20,000 SEK. Varying second-line treatment costs by ±50% had little effect on the ICER. Reducing the proportion of patients that required second-line treatment to 0% reduced the ICER by approximately 20%. The inclusion of additional therapeutic drug monitoring for each treatment only reduced the ICER by approximately 2000 SEK. When assuming that 100% of patients began on IV treatment, the ICER per additional QALY gained was 190,397 SEK. When the life expectancy was halved, the ICER was almost doubled, while increasing the life expectancy by 50% reduced the ICER by a quarter. However, in all scenarios tested, ICER remained cost-effective.

**Table 5:** Scenario sensitivity analyses

Parameter	Evaluated parameter	ΔCosts (SEK)	ΔEffects (QALYs)	ICER (costs per QALY gained)
Mucormycosis prevalence	14%	59,191	0.72	81,464
Pathogen identification percentage	0%	64,793	0.34	189,966
	100%	39,588	0.26	154,784
Second-line treatment drug costs	−50%	53,701	0.3	179,950
	+ 50%	50,681	0.3	169,830
Percentage receiving second-line treatment	0%	42,361	0.3	141,952
Percentage requiring therapeutic drug monitoring	50% for isavuconazole and 75% for voriconazole/posaconazole	51,661	0.3	173,115
Percentage starting with IV formulation	100%	56,818	0.3	190,397
Life expectancy	−50%	52,191	0.17	310,924
	+ 50%	52,191	0.40	130,484

Δ, difference, *ICER* incremental cost-effectiveness ratio, *IV* intravenous, *QALY* quality-adjusted life year, *SEK* Swedish krona

## Discussion

This model was developed to assess the cost-effectiveness of isavuconazole from a Swedish healthcare payer perspective for the treatment of possible IA in adult patients when, at the point of treatment initiation, a differential diagnosis between IA and mucormycosis had not been achieved. Isavuconazole was found to be cost effective at a WTP threshold of 1000,000 SEK and had an ICER of 174,890 SEK per additional QALY gained. Data for between 2005 and 2011 for TLV decisions on the inclusion of drugs in the pharmaceutical benefits scheme show that the lowest cost per QALY declined was 700,000 SEK while the highest accepted was 1,220,000 SEK [35]. This compares favourably also to oncology drugs that have been assessed positively by the Swedish council for novel therapeutics with ICERs per QALY up to 1,080,000 SEK (with pertuzumab being the exemption, having an ICER at 2,600,000 SEK) [36].

The higher QALYs in the isavuconazole arm than in the voriconazole arm of the study were mainly attributable to the lack of effect of voriconazole for the treatment of mucormycosis. Isavuconazole is active against both *Aspergillus* and Mucorales, and may be advantageous compared with voriconazole in patients with possible IA in whom mucormycosis has not been ruled out. Patients receiving voriconazole in this model had, on average, increased mortality, as a proportion either received ineffective treatment, or experienced a delay in receiving effective treatment before pathogen information became available, which is reflective of real world situations. In a study of 230 cases of mucormycosis in Europe, 48% of patients were receiving antifungal drugs, including voriconazole and caspofungin, prior to diagnosis being confirmed [37]. Similarly, in a multicentre cohort study of patients with mucormycosis after haematopoietic stem cell transplantation, 29 cases were identified, of which 23 were receiving antifungal treatment at diagnosis, of which half were receiving voriconazole. Differential diagnosis between the two pathogens is important, as untreated mucormycosis has a mortality rate approaching 100% [13] and a delay in treatment of up to 6 days has been demonstrated to result in a near doubling of the rate of mortality (83% vs 43%) [10]. However, the absence of a differential diagnosis between IA and mucormycosis can be a challenge in real-world practice where treatments have to be administered before diagnosis, unless the initial treatment has efficacy against both IA and Mucorales. In fact, the selection of approximately 6% as the percentage of patients who actually had mucormycosis in the current study, which was based on hospital diagnosis data (International Classification of Diseases-10 [ICD-10]) from France [3], may have been overly conservative, because the prevalence of mucormycosis among patients with invasive mould infections has been reported to be as high as 14% in a Swedish cohort of patients [9] or 9% in a study of 6807 patients with IFD in North American health centres [38]. Surveillance studies from transplant centres in the US have reported prevalence data for mucormycosis relative to all patients with IA or mucormycosis of between around 9% [39] and 16% [40]. On the other hand, prevalence may vary considerably by region and by study, as relative prevalence estimates from Italian studies have ranged from around 4% [41] to 10% [42]. The model is robust for a wide range of mucormycosis prevalence (4 to 14%, tested from the deterministic sensitivity analysis [DSA] and Scenario analyses).

In clinical practice, differential diagnosis is difficult to make and often patients can be infected with multiple pathogens; in a retrospective observational study of invasive mould disease in a single centre in Sweden, multiple mould pathogens were observed in 13% of disease [9]. In the SECURE study, only 53% of patients had pathogen information available (proven and probable cases) and only 13% of patients had proven invasive mould disease [11]; in a German study of 170 patients evaluating the European Organisation for Research and Treatment of Cancer and the National Institute of Allergy and Infectious Diseases Mycology Study Group (EORTC/MSG) criteria for IFD, only 14% of patients had pathogen information available (proven and probable cases) and only 3% had proven IFD.

Cost-minimization studies comparing isavuconazole with L-AmB followed by posaconazole for the treatment of invasive aspergillosis and mucormycosis have shown estimated treatment costs to be at least 20% lower with isavuconazole in a United Kingdom (UK) healthcare setting [21, 43]. Similarly, in the treatment of mucormycosis, cost savings relative to L-AmB have been demonstrated for isavuconazole in German and Italian healthcare settings [44, 45].

The model is limited by the published data used as inputs in the model. Data from the SECURE and VITAL trials were based on international populations of patients and not localised data for Sweden. However, the sensitivity analyses in this model allowed for a range of assumptions to be analysed. It could not always be confirmed that data from literature or international databases used in the model were relevant to Sweden. Nevertheless, they were clinically plausible and there was no reason to believe that they should not be applicable. Furthermore, assumptions regarding the duration of some treatments, although plausible, were made in line with separate cost-effectiveness analyses. Life expectancy in the model was based on that of patients with AML. Although AML was the dominant underlying condition observed in the SECURE trial, it still accounted for less than 50% of the total patients. Varying life expectancy by 50% had a measurable effect on the model, but the scenario sensitivity analysis showed that a cost-effective ICER was still provided.

In the current model, we captured only two specific AEs due to their suspected economic impact, whereas the SECURE trial indicated notable differences between isavuconazole and voriconazole in the incidence of other AEs as well [11]. This difference may be because therapeutic drug monitoring of voriconazole was not performed in the SECURE trial. Furthermore, the rates of 30-day re-admissions and rate of serious AEs post-discharge leading to re-hospitalisation were numerically higher with voriconazole [22]. Thus, the conservative approach taken in the current analysis is likely to have underestimated the differences between both drugs. Isavuconazole also has a lower predisposition than voriconazole for drug-drug interactions with immunosuppressant drugs [46]. However, the effects of drug-drug interactions on patient and cost outcomes in IFD patients have not been reported and hence these potential differences could not be captured. Differences in treatments for specific subpopulations of patients were not captured in the model, particularly in subpopulations where voriconazole treatment would not have been suitable, such as patients with QT prolongation, severe renal impairment, or concomitant treatment with drugs with which interactions would have been an issue [18].

## Conclusions

This study is the first cost-effectiveness analysis of isavuconazole compared with voriconazole in the treatment of possible IA.

Overall, from a Swedish healthcare payer perspective, this model suggests that treatment of possible IA with isavuconazole is cost-effective compared with voriconazole in adult patients due to the additional Mucorales coverage provided by isavuconazole.

## Additional files

Additional file 1: **Table S1.** Outpatient monitoring costs. Table summarising the outgoing monitoring costs of patients (DOCX 13 kb)

Additional file 2: **Table S2**. Distributions used in the probabilistic sensitivity analysis. Table summarising the probabilistic variables and their distributional parameters used in the probabilistic sensitivity analysis (DOCX 14 kb)

Additional file 3: **Table S3**. Parameters tested in deterministic sensitivity analysis. Table summarising the parameters tested in the deterministic sensitivity analysis (DOCX 13 kb)

## Abbreviations

AE: Adverse event
AmB: Amphotericin B
AML: Acute myeloid leukaemia
DSA: Deterministic sensitivity analysis
EMA: European medicines agency
EORTC: European organization for research and treatment of cancer
FDA: Food and drug administration
HSCT: Haematopoietic stem cell transplant
IA: Invasive aspergillosis
ICD: International classification of diseases
ICER: Incremental cost-effectiveness ratio
IFD: Invasive fungal diseases
IV: Intravenous
L-AmB: Liposomal amphotericin B
MSG: Mycology study group
QALY: Quality adjusted life-year
SD: Standard deviation
SEK: Swedish krona
SmPC: Summary of product characteristics
TEAE: Treatment-emergent adverse event
TLV: Tandvårds- och läkemedelsförmånsverket
UK: United Kingdom
US: United States
WTP: Willingness to pay
