# Supplementary material for: Cost-effectiveness analysis of isavuconazole versus voriconazole for the treatment of patients with possible invasive aspergillosis in Sweden
Source: BMC Infect Dis. 2019 Feb 11;19:134. doi: 10.1186/s12879-019-3683-2 (PMC6371439; doi:10.1186/s12879-019-3683-2)
Supplement: Supplementary file 1 — Table S1. Outpatient monitoring costs. Table summarising the outgoing monitoring costs of patients (DOCX 13 kb) [file 12879_2019_3683_MOESM1_ESM.docx]

**Table S1**. Outpatient monitoring costs

|  | **Isavuconazole** | **Voriconazole** | **L-AmB to posaconazole** | **L-AmB to voriconazole** |
| --- | --- | --- | --- | --- |
| Treatments in IA  Outpatient days, n  Liver function test, n | 60.8  4 | 60.8  7 | 27.4  4 | 27.4  4 |
| Treatments in mucormycosis  Outpatient days, n  Liver function test, n | 199  14 |  | 122  17 |  |

IA, invasive aspergillosis; L-AmB, liposomal amphotericin-B
