# Supplementary material for: Cost-effectiveness analysis of isavuconazole versus voriconazole for the treatment of patients with possible invasive aspergillosis in Sweden
Source: BMC Infect Dis. 2019 Feb 11;19:134. doi: 10.1186/s12879-019-3683-2 (PMC6371439; doi:10.1186/s12879-019-3683-2)
Supplement: Supplementary file 2 — Table S2. Distributions used in the probabilistic sensitivity analysis. Table summarising the probabilistic variables and their distributional parameters used in the probabilistic sensitivity analysis (DOCX 14 kb) [file 12879_2019_3683_MOESM2_ESM.docx]

**Table S2**. Distributions used in the probabilistic sensitivity analysis

| **Type and properties of distribution** | **Parameter** | **Mean value (SE)** |
| --- | --- | --- |
| Gamma: Bounded at 0, positively skewed. Derived from mean and its SE. SE was set at deterministic cost/4. Alpha and Beta values were calculated as follows: Alpha = (mean/SE)2 Beta = SE2/Mean | Costs |  |
|  | Isavuconazole – IV | 5,311 (1,328) |
|  | Isavuconazole – oral | 7,067 (1,767) |
|  | Voriconazole – IV | 1,167 (292) |
|  | Voriconazole – oral | 8,008 (2,002) |
|  | Amphotericin B – IV | 15,869 (3,967) |
|  | Posaconazole – oral | 27,938 (6984) |
|  | Liver function tests^a^ | 611 (153) |
|  | Therapeutic drug monitoring | 1,316 (329) |
|  | Cardiac arrest | 43,228 (10,807) |
|  | Tachycardia | 8,976 (2,244) |
|  | Hepatologic disorders | 3,863 (966) |
|  | Hospitalisation costs (hematology) | 7,006 (1,752) |
|  | Hospitalisation costs (renal) | 9,924 (2,481) |
|  | Life expectancy | 17 (4.25) |
| Beta: Bounded between 0 and 1. As the sample size and the number of events were specified Alpha and Beta values were calculated accordingly | Percent requiring 2^nd^line treatment |  |
|  | IA | 47.76 (3.65) |
|  | Mucormycosis | 33.33 (28.42) |
|  | Mortality |  |
|  | IA | 29.07 (2.05) |
|  | Mucormycosis | 43 (0.4) |
|  | Mortality with delayed treatment | 83 (1.87) |
|  | Mortality with untreated disease | 96 (0.01) |
|  | Quality of life | 0.82 (0.02) |

IA, invasive aspergillosis; SE, standard error
