# Supplementary material for: Cost-effectiveness analysis of isavuconazole versus voriconazole for the treatment of patients with possible invasive aspergillosis in Sweden
Source: BMC Infect Dis. 2019 Feb 11;19:134. doi: 10.1186/s12879-019-3683-2 (PMC6371439; doi:10.1186/s12879-019-3683-2)
Supplement: Supplementary file 3 — Table S3. Parameters tested in deterministic sensitivity analysis. Table summarising the parameters tested in the deterministic sensitivity analysis (DOCX 13 kb) [file 12879_2019_3683_MOESM3_ESM.docx]

**Table S3**. Parameters tested in deterministic sensitivity analysis

| **Parameter** | **Change** |
| --- | --- |
| Voriconazole IV price | −25%, +25% |
| Voriconazole oral price | −25%, +25% |
| Percent of patients requiring second-line treatment - IA | −25%, +25% |
| Percent of patients requiring second-line treatment - mucormycosis | −25%, +25% |
| Mortality - isavuconazole IA | −25%, +25% |
| Mortality – isavuconazole mucormycosis | −25%, +25% |
| Mortality – delayed treatment | −30%, +20% |
| Mortality – untreated | −30%, +4% |
| Quality of life estimate | −20%, +20% |
| Life expectancy | −25%, +25% |
| Treatment duration – isavuconazole – invasive aspergillosis | −25%, +25% |
| Treatment duration – isavuconazole – invasive aspergillosis (prior to switching) | −25%, +25% |
| Treatment duration – isavuconazole – mucormycosis | −25%, +25% |
| Treatment duration – voriconazole – mucormycosis | −25%, +25% |
| Mucormycosis prevalence | −25%, +25% |
| Pathogen identification information percentage | −25%, +25% |

IA, invasive aspergillosis; IV: intravenous
